# Functional Liver Cell–Based Platforms in Biomedical Research

**DOI:** 10.1002/prp2.70128

**Published:** 2025-06-02

**Authors:** Zohreh Hashemian, Sara Taleahmad, Bahare Shokouhian, Mustapha Najimi, Massoud Vosough

**Affiliations:** ^1^ Department of Regenerative Medicine, Cell Science Research Center Royan Institute for Stem Cell Biology and Technology, ACECR Tehran Iran; ^2^ Department of Applied Cell Sciences, Faculty of Basic Sciences and Advanced Medical Technologies Royan Institute, ACECR Tehran Iran; ^3^ Department of Stem Cells and Developmental Biology, Cell Science Research Center Royan Institute for Stem Cell Biology and Technology, ACECR Tehran Iran; ^4^ Department of Medical Biotechnology, School of Advanced Technologies in Medicine Tehran University of Medical Sciences Tehran Iran; ^5^ Laboratory of Pediatric Hepatology and Cell Therapy Institute of Experimental and Clinical Research (IREC), UCLouvain Brussels Belgium; ^6^ Experimental Cancer Medicine Institution for Laboratory Medicine, Karolinska Institute Stockholm Sweden

**Keywords:** biomedical research, drug screening platforms, hepatocytes, hepatoma cells, improved metabolic performance

## Abstract

Recapitulating in vivo conditions of metabolism remains a challenging subject in biomedical research such as ADME‐Tox assays (absorption, distribution, metabolism, excretion, and toxicity). The advanced technologies using 3D co‐culture methods enabled researchers to develop cell–cell and cell–extracellular matrix (ECM) interactions similar to the natural liver, resulting in the improvement of the metabolic performance of ex vivo cultured primary hepatocytes (PHs). Although PHs are the best candidates in cell‐based drug screening methods, access to these cells is limited. The application of stem cell–derived hepatocyte‐like cells (HLCs) could overcome these limitations in high‐throughput assessments. However, the functional capacity of HLCs is not enough. Hepatoma cells could be reliable substitutes for PHs and HLCs; however, compared to PHs, their metabolic performance is low. Mimicking the complexity of the liver microenvironment using hepatoma cells and liver‐specific stromal cells in a 3D culture condition represents an innovative, accessible, and scalable platform to accelerate drug development if the metabolic capacity of hepatoma cells is enhanced. This can reduce time, costs, and address the ethical concerns related to animal models and pluripotent stem cells. In this manuscript, we showed that mimicking the complexity of the liver microenvironment in a 3D co‐culture condition with non‐parenchymal cells and improving the metabolic performance of hepatoma cells represents an innovative and accessible platform to accelerate drug discovery and development.

## Graphical abstract

Exploring various sources for hepatocytes: Advantages and disadvantages of each source of cells; foundations for advancements in the ADME‐Tox assay.

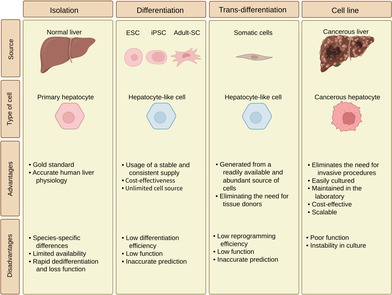

**Abbreviations**
2D: two‐dimensional
3D: three‐dimensional
ADME‐Tox: absorption, distribution, metabolism, excretion, and toxicity
DILI: drug‐induced liver injury
ECM: extracellular matrix
HLC: hepatocyte‐like cell
iPSC: induced pluripotent stem cell
PH: primary hepatocyte
RUCAM: Roussel Uclaf causality assessment method

## 1 Introduction

Nowadays, chronic degenerative diseases and age‐related disabilities are the main concerns for health experts. This situation necessitates proposing innovative modalities in drug development. Novel medications application should be validated and approved in lab‐ and animal model‐based platforms. Considering that hepatic cells are in charge of metabolism, activation, and breakdown of the majority of medicines and xenobiotics, primary hepatocyte (PH)–based platforms are the best tools in this regard. However, the main challenge in the broad application of these platforms is the inadequate supply of functional hepatocytes [1, 2, 3].

ADME‐Tox assays, which include the assessment of absorption, distribution, metabolism, excretion, and toxicity, are an essential part of the drug development procedure. These analyses provide essential data regarding how a candidate drug engages with metabolic pathways in the human body. This can guide researchers for optimized decisions at every stage of the drug development process [4]. These assessments not only help to figure out the bioactive drug uptake and its entry into the circulatory system but also how it may influence the liver. Since the distribution of the compound within different tissues, especially hepatocyte cells, is considered a very important factor in measuring the risk of drug‐induced liver injury (DILI), it is crucial to understand how the compound distributes. Also, metabolism tests and knowledge of biotransformation pathways help in understanding drug and protein interactions and the mechanism of DILI. Excretion assessments on how the body removes the drug and its possibly toxic metabolites suggest critical understanding into the risk of hepatotoxicity. Toxicity appraisals play a substantial role in addressing safety concerns, measuring the possible side effects and genotoxicity. On the other hand, these assays provide results for smart decisions based on safety, efficiency, and hepatotoxicity [5, 6, 7].

Hepatocyte‐based platforms play a central role in the thorough evaluation of ADME‐Tox in the drug development industry. The liver is the first site of drug detoxification and works as a drug biotransformation factory [8, 9]. Using liver‐forming cells, especially hepatocytes, in in vitro evaluations enables researchers to imitate the complicated processes of drug metabolism in vivo. These provide data on a drug candidate for evaluation of possible metabolites and the risk of toxicity [7, 10]. Hepatocyte‐based evaluations are important in apprising drug‐caused liver toxicity, an extremely important part of ADME‐Tox. By treating hepatocytes with new drug candidates, scientists can identify possible harmful effects on liver cells, and enabling them to detect compounds contributing to hepatotoxicity at an early stage. Basically, hepatocyte‐based evaluations serve as extremely valuable tools for the safety of drugs in the body, enabling the selection of talented candidates. While these tests reduce the risk of hepatotoxicity [8, 11, 12].

DILI and ADME‐Tox testing are two key areas in drug discovery and clinical hepatology. DILI refers to the actual and clinical occurrence of liver injury after drug administration, which is usually detected by enzyme tests (ALT, AST), histopathological evaluation, and tools such as the Roussel Uclaf causality assessment method (RUCAM). In contrast, ADME‐Tox tests are prospective preclinical tools designed to predict the risk of toxicity, including DILI, before a drug enters human trials [13].

The mechanism of DILI involves two major pathways: direct cellular damage (intrinsic toxicity), exemplified by acetaminophen overdose and the production of the toxic metabolite NAPQI, and idiosyncratic reactions, dependent on genetic background (e.g., HLA haplotypes) and delayed immune responses. Considering the “law of two,” drugs with high lipophilicity (logP ≥ 3) and daily doses greater than 100 mg are more likely to cause DILI [14].

ADME‐Tox assays play a critical role in risk reduction by assessing drug absorption, distribution, metabolism, excretion, and toxicity. These assays include models such as 3D hepatocytes, high‐content imaging for early detection of damage (steatosis, phospholipidosis, lysosomal entrapment), and liver‐on‐a‐chip systems that are capable of reproducing chronic and immune‐mediated toxicity. These innovative models have achieved high predictive accuracy (up to 85%) and even outperform animal models [15] (Table 1).

**TABLE 1:** Comparison of DILI and ADME‐Tox assays.

Aspect	Test	
	DILI	ADME‐Tox
Definition	Clinical liver injury caused by drugs/toxins	In vitro/in silico safety prediction assays
Purpose	Diagnosis and characterization	Predict and prevent toxicity preclinical
Timing	After drug exposure	Before human exposure
Methodology	Enzyme tests (ALT, AST), RUCAM, histology	3D liver models, omics, high‐content imaging
Key mechanisms	Intrinsic (e.g., NAPQI) or immune‐mediated	P450 metabolism, lysosomal/membrane effects
Clinical use	Used in diagnosis and management of liver injuries	Decisions guide in drug development

This article highlights the confronted challenges in drug discovery and demonstrates cutting‐edge approaches. We discuss novel approaches in the pharmaceutical and biomedical research landscape that can lead us to more effective and safer screening methods using hepatocyte‐based platforms.

## 2 Diverse Hepatocytic Cells in Drug Screening

### 2.1 Primary Hepatocytes

**FIGURE 1:**
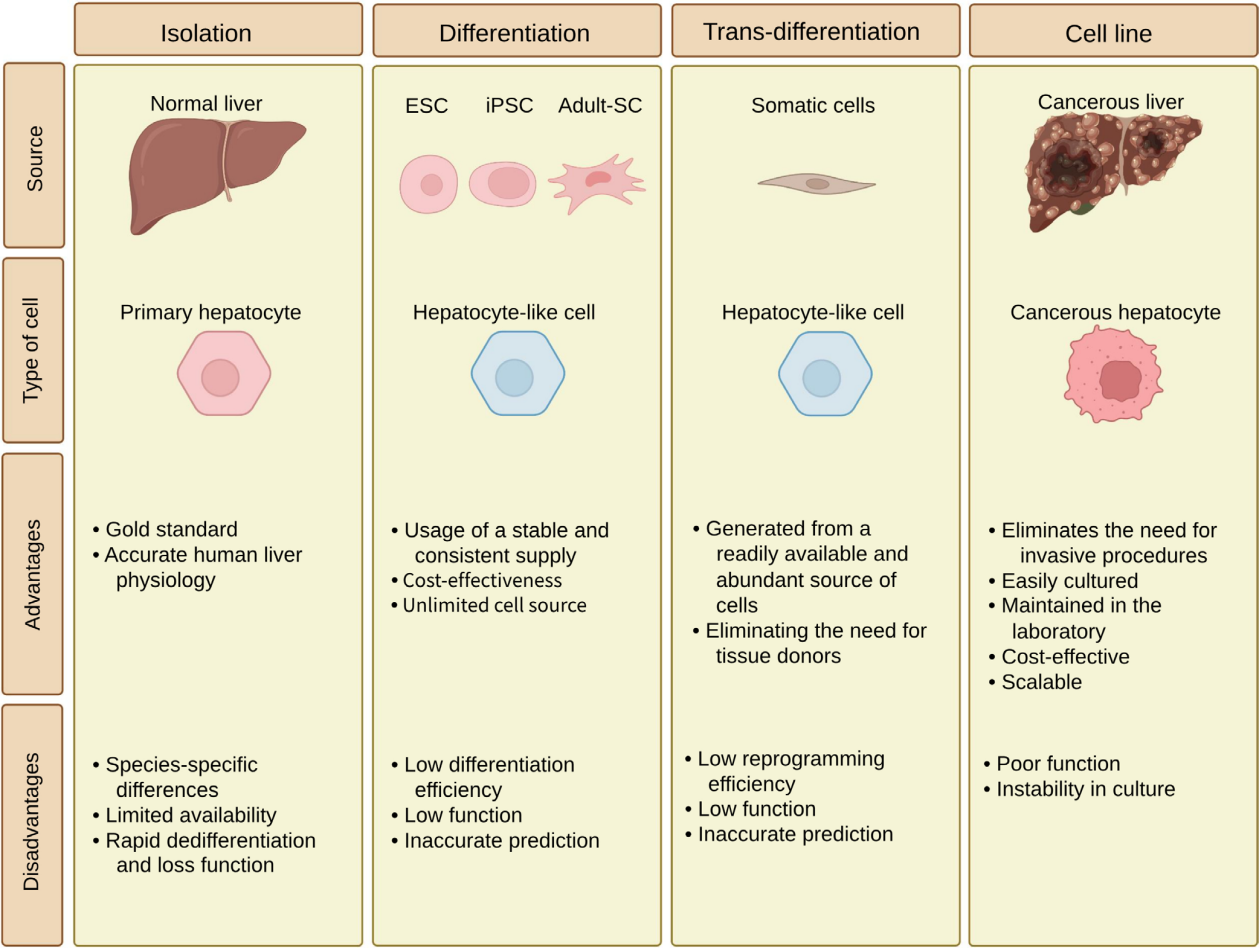
Exploring various hepatocyte sources: foundations for advancements in the ADME‐Tox assay.

PHs are a gold standard tool for drug screening and toxicity testing [16]. Isolation of PHs directly from the liver preserves many inherent hepatic features [17]. The use of PHs enables researchers to evaluate the impact of drugs within a biologically relevant context, as they retain vital metabolic pathways, drug‐processing enzymes, and transporter proteins [18]. However, challenges are present in availability, long‐term culturing, and sustaining the functionality of PHs under ex vivo condition, as they tend to dedifferentiate over time. PHs undergo significant functional and morphological changes after 3–5 days of in vitro culture under 2D monolayer conditions. In the initial hours, the expression of liver‐specific genes such as *ALB* and *FOXA2*, and drug‐metabolizing enzymes, such as CYP1A2 and CYP7A1, are significantly reduced. By Day 3, hepatocytes lose their polygonal morphology and transform into flat shapes with weak cell–cell boundaries. By Day 5, the hepatocyte architecture is completely destroyed and signs of epithelial‐to‐mesenchymal transition (EMT) are observed [19]. In spite of these challenges, PHs are considered the gold standard tool for evaluating the in vitro effects of and potential liver toxicity of drug candidates in the early stages of preclinical drug development (Figure 1) [20, 21].

### 2.2 Pluripotent Stem Cells (PSCs)

Obtaining hepatocyte‐like cells (HLCs) through the directed differentiation of stem cells has been proposed as a new and innovative platform for drug screening. This approach has provided a possibly renewable source of cells. However, ethical challenges in embryonic stem cell usage and the complexity of differentiation procedures makes it difficult for use in clinical applications [22]. However, the advantages are amazing and interesting; these HLCs can overcome certain restrictions connected to the PHs, as they can be customized to match specific requirements and to provide a consistent source of cells for long‐term research studies. HLCs have become promising substitutes for in vitro drug screening, disease modeling, and applications in decorated personalized medicine (Figure 1) [23, 24]. Stem cell–derived cells are called “HLCs” due to their functional and molecular limitations reported in various studies. These cells have immature characteristics, including transcriptional profiles that are similar to embryonic cells and the absence of mature markers such as CYP2C9 and CYP3A4. HLCs also have functional defects, such as low albumin secretion and lower urea production than PHs. Incomplete differentiation has also been observed in these cells; for instance, some of them express markers of other cells such as intestinal or fibroblastic cells. Their metabolic capacity is also limited, especially in phase II, and their ability to predict hepatotoxicity is limited. Technically, differentiation protocols are variable and the metabolic function of HLCs decreases after a short period of time [25, 26, 27].

### 2.3 Induced Pluripotent Stem Cells (iPSCs)

Induced pluripotent stem cells (iPSCs) provided a reliable cell source for producing HLCs for drug screening and toxicology [28]. IPSCs are generated through the reprogramming of somatic cells with no ethical concerns. Besides, challenges in this approach include the complexities of the reprogramming process and inducing complete differentiation in cells. If the current challenges are addressed, researchers will have a powerful tool for studying the molecular and cellular mechanisms of diseases and personalized drug responses (Figure 1) [29, 30].

### 2.4 Adult Stem Cells

Adult stem cells originate from various tissues all over the body. These cells can be an alternative source for generating HLCs by re‐differentiation [22]. They provide a balance between ethical considerations, accessibility, and the requirement for a stable and flexible in vitro assessment for drug screening and hepatotoxicity. Similar to hepatocytes derived from other stem cell sources, the main challenges include not being able to replicate completely the complexity of PHs (Figure 1) [16].

## 3 History of Hepatocyte‐Based Platforms in Drug Screening

The application of liver cells in drug screening and toxicology has gradually increased over the years. Scientists have generated more effective modalities to improve hepatic metabolism for drug discovery studies [28, 29].

The 2D PH cultures have been used since the 1980s. These systems worked for studies on drug metabolism; however, the major limitation of 2D cultures was the rapid loss of liver‐specific functions due to de‐differentiation of hepatocytes, such as drug‐metabolizing enzyme activity. Lack of cell–cell and cell–ECM interactions results in de‐differentiation of ex vivo liver cells [28, 29].

**FIGURE 2:**
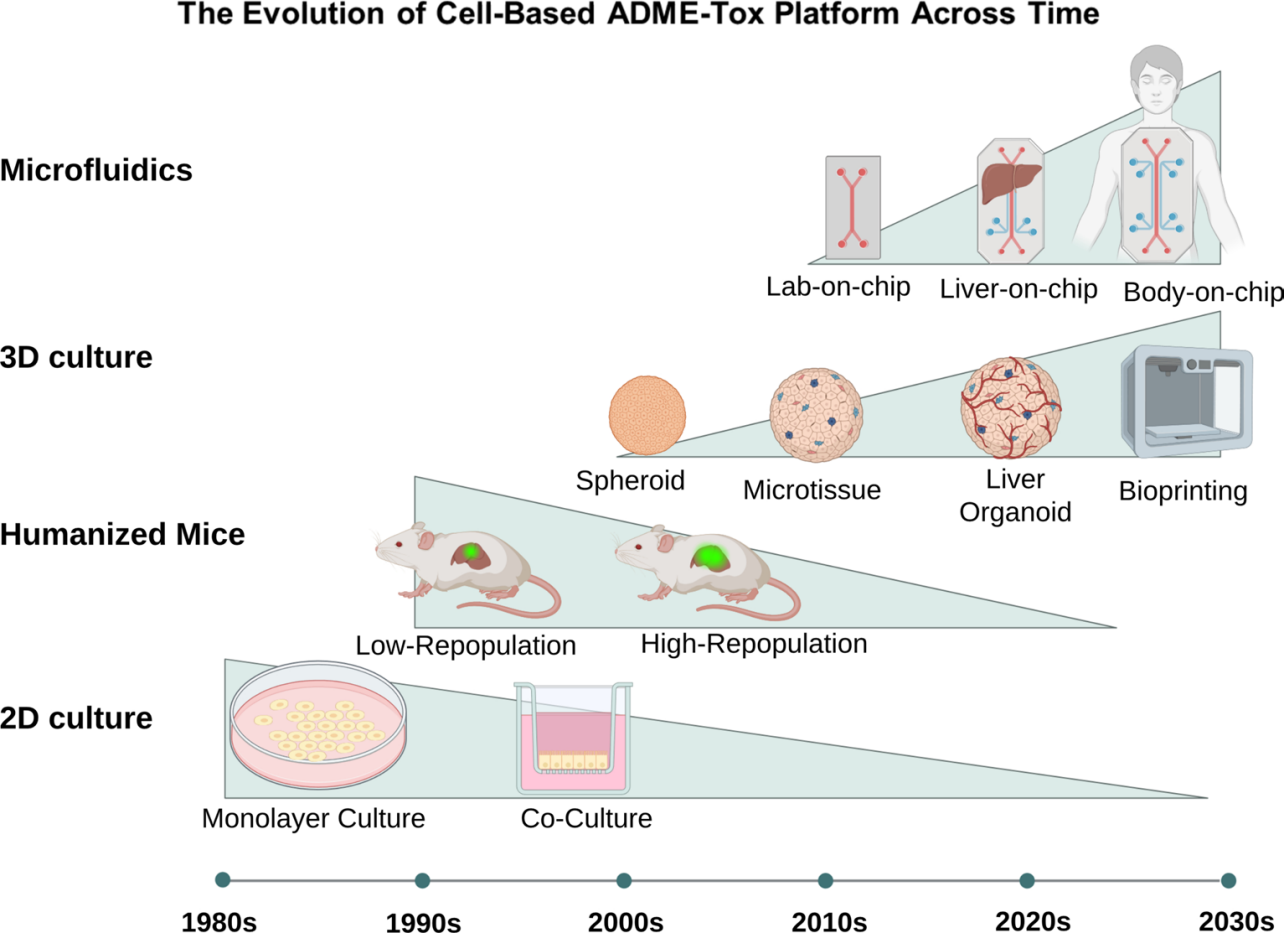
The trend of using different culture configurations based on hepatocytes in drug screening and toxicology. In the early 1980s, 2D culture systems in monoculture and co‐culture were very common; however, emerging novel methods gradually decreased their applications. Humanized mice were created and adopted in the 1990s and their chimeric percentage gradually increased; however, their broad application is decreasing due to ethical issues. 3D culture began in the early 2000s in simple formats and gradually expanded to incorporate different cell types. It is now increasingly being developed as an advanced model for various microtissues. In the 2010s, microfluidic systems were developed. Recent progresses have led to advanced technologies such as organ‐on‐chip and human‐on‐chip.

To overcome the limitation of 2D cultures, scientists began to develop three‐dimensional (3D) hepatocyte cultures in the early 2000s. These culture systems are called liver microtissues, spheroids, organoids, and bio‐printed tissues. These models mainly mimic the structure and physiology of the liver by providing cell–cell and cell–ECM interactions, and they maintain hepatocyte functions for a longer period of time. These platforms provide more realistic drug metabolism studies by using hepatocytes in a liver‐like microenvironment [30, 31]. Since the early 2010s, hepatocytes have been used in “liver‐on‐a‐chip” devices. In these micro labs, 3D hepatic microtissues were incubated in a dynamic state in flowing media, mimicking blood supply in the liver. This approach enabled researchers to have more accurate studies on drug metabolism and safety [32]. Since the 1990s, mice with humanized livers have been introduced. Human liver cells are transplanted into a mice model, and the mouse liver is replenished with human liver cells. In the 2000s, this method became an important platform to study human‐specific drug metabolism and toxicity in vivo. Despite its significant potential in drug development and disease modeling, organ‐on‐chip technology faces some challenges such as material limitations (e.g., drug uptake by PDMS), lack of standardization, technical difficulties (e.g., multi‐organ integration and limited stability), and high costs. In addition, the lack of physiological complexities and limitations in sensor integration limits the ability to model complex diseases. Future innovative directions, including the use of novel materials, AI‐based design, and global efforts for standardization, could improve this technology from being a limited research tool to a widely used platform in drug development [33]. These achievements in the field of drug screening have helped a lot in the direction of developing safer and more effective drugs and accelerating their transfer from the bench to the bedside. Research in this field continues to generate more realistic models of normal and specific liver diseases (Figure 2).

Comparisons costs between these configurations, 2D cultures are reasonable, but they lack physiological relevance. Humanized mice models are very expensive (including genetic engineering costs and long‐term animal care); however, they can provide more in vivo relevance, which is critical for drug development studies. 3D cell cultures are more expensive due to the use of specialized matrices, and they provide more physiological biomimics. Microfluidic devices come in a variety of prices based on the complexity of their design. Microfluidic systems created with modern 3D printers are less expensive than microfluidic systems designed with traditional photolithography techniques due to their speed and customizable features [34, 35, 36].

## 4 Hepatocytes in Various Culture Configurations for Drug Screening

### 4.1 2D Culture and Developed Platforms for Commercialization

The cells used in hepatocyte‐based drug screening and hepatotoxicity assessments are divided into three general categories: PHs, stem cell‐derived HLCs, and hepatoma cell lines [16, 28]. PHs are isolated from donated liver. Now they are accepted as a standard tool for in vitro studies. They closely imitate the performance of in vivo hepatocytes and offer a reliable platform for drug screening and hepatotoxicity tests. Therefore, several companies like Lonza, Gibco, Ixcells Biotech, Corning, and BioPridict International have commercialized PH‐derived tools from human cells and different animal sources [32, 34, 37, 38, 39].

HLCs are derived from pluripotent stem cell sources, including iPSCs and ESC. Therefore, some biotech companies have provided hepatocyte progenitor cells and HLCs, such as Fujifilm (iPSC‐derived hepatocytes), ATCC (ATCC‐HYS0103‐iPSC‐derived hepatocytes), and Thermo Fisher Scientific (HepaRG—a human immortalized hepatic cell line) [34, 39, 40]. HepaRG cells have been identified as a promising alternative cell to primary human hepatocytes in drug metabolism and toxicity studies. After differentiation, these cells exhibit enzymatic activities similar to PHs and are a better option for drug toxicity tests compared to HepG2. HepaRG cells also perform better metabolic function in 3D spheroid models, including having more stable CYP3A4 activity and enhanced drug metabolism. However, there are limitations such as an unusual response to some hepatotoxins and a lack of complete replication of hepatocyte complexity. These cells are useful for applications such as high‐throughput drug screening and chronic toxicity studies, but they require standardization of protocols for optimal application [41, 42]. More hepatoma cell lines, such as Huh‐7, Hep3B, and HepG2, are of human hepatocellular carcinoma origin [43, 44]. However, some hepatocyte cell lines have their origin from normal hepatocyte cells such as Chang liver cells (normal human hepatocytes), L‐O2 (human fetal hepatocyte), AMl‐12 (normal human hepatocyte) [45, 46]. Also, non‐parenchymal cell lines such as LX2 cells have been established. LX2, a hepatic stellate cell line, is used in drug screening studies for fibrosis [47, 48]. Liver cell lines are commonly used in drug screening tests for their easy access, maintenance, and high proliferation. Several suppliers, including ATCC, the European Collection of Authenticated Cell Cultures (ECACC), and the Japanese Collection of Research Bioresources Cell Bank (JCRB), offer these cell lines for research purposes. The choice of cell type in drug screening tests depends on different aspects such as specific research goals, balancing physiological relevance, ease of use, cost‐effectiveness, and availability [34, 40, 49, 50, 51].

### 4.2 3D Culture Models and Advanced Platforms for Commercialization

Three‐dimensional (3D) culture systems have emerged as a promising alternative modality to traditional 2D models, marking a significant progress in the field of tissue engineering. There are two main approaches to 3D cell culture: spheroids and microtissues. Spheroids are cell aggregations that can mimic some aspects of a tumor environment. The use of Spheroids is simple and cost‐effective, but comes with challenges like limited lifespan, having poor nutrient and oxygen flow in their cores. Microtissues approaches use various materials such as hydrogels and electrospun fibers besides cell types. Exciting new tools, including 4D bioprinting and hybrid scaffolds, are pushing the field forward. These innovations hold promise for better replicating human biology, which could ultimately make drug screening and disease research more reliable [29, 52]. By embedding hepatocytes within matrices that imitate the liver's ECM, these systems enable the hepatocytes to self‐organize into hepatic‐like structures that mimic the liver microenvironment. The early developments of this technology started with the introduction of spheroid cultures in the 1970s [49, 50, 53]. These cell aggregates provide natural interactions among hepatocytes and maintain liver‐specific functions for extended periods of time compared to traditional 2D culture methods. By improving these systems, spheroid cultures strengthen the foundation for subsequent sophisticated 3D culture configurations. 3D culture conditions simulate the liver's natural microenvironment and its complex functions [51, 54, 55].

Recently, further high‐quality 3D culture models use co‐culture systems to improve the interactions between hepatocytes and non‐parenchymal liver cells. In 2010s, 3D bioprinting technology made a remarkable progress in the field of liver organoids [45, 56, 57]. Organoids provide a miniature version of the liver, earning its complex architecture and multicellular pattern and biochemical dynamics. At the same time, bioprinting offers exact control in engineering tissue structures, layer by layer. These 3D culture models provide an extremely valuable platform, especially for the study of drugs with complex metabolic profiles. They enable a fidelity to in vivo responses that is unparalleled in 2D assays, making them an essential part of increasing the precision and predictive power of preclinical research [45, 48]. 3D bioprinting technology has revolutionized drug screening methods. Using liver tissues that are similar to human tissues, it has become possible to test drugs more accurately and without the need to use laboratory animals. These tissues are made from the patient's own cells and help analyze individual responses to drugs. Using artificial intelligence and advanced equipment, the accuracy and efficiency of these models have increased significantly. These methods can reduce the time and cost of drug production in the future and help advance more precise and safer medicine [58].

Commercial 3D culture systems based on hepatocytes, which include organoids and microtissues, are designed to closely mimic the hepatic environment and function. Organovo is a company that specializes in bioprinting technology, and their ExVive human liver tissues are 3D bioprinted structures that mimic the histological architecture of the human liver [59]. InSphero company provides InSphero 3D InSight, spheroid‐based microtissues that contain primary human hepatocytes co‐cultured with liver non‐parenchymal cells, which offer a more physiologically relevant model for drug discovery and safety assessments [60]. Ascendance Biotechnology produce HepatoPac and HepatoMune. HepatoPac is a micro‐patterned co‐culture of PHs and stromal cells that generates a “micro‐liver” useful for metabolism, safety testing, and disease modeling. HepatoMune is a similar system optimized for inflammatory status and immune‐mediated studies of the liver [61].

These commercial models are useful tools in drug development pipelines, enabling researchers to study hepatic function, drug metabolism, toxicity, and disease modeling in an in vitro setting that imitates the in vivo human liver.

### 4.3 Microfluidics and Developed Platforms for Commercialization

Microfluidic devices have made significant developments in hepatocyte‐based drug screening technologies, combining accurate control over culture conditions with dynamic factors such as perfusion rate, oxygen gradients, and shear stress to closely imitate those of the liver's in vivo conditions. Since the early 2000s, the field of liver microfluidics has been taking advantage of rapid technological progress to study hepatocyte interactions within the big dynamic cellular picture. By the mid‐ to late 2000s, these microfluidic systems began incorporating liver cells, giving rise to early liver‐on‐a‐chip models that aimed to imitate liver functions on a microscale. This progress enabled researchers to monitor drug metabolism and distribution within the body, providing a proper platform for drug pharmacokinetics studies [62, 63, 64, 65].

Development of this technology reached a turning point in 2013 when “human‐on‐a‐chip” devices were introduced as connected multiple organs. Over the past 10 years, liver‐on‐a‐chip technologies have grown much more advanced, with current systems using 3D cell cultures and different cell types to mimic the liver's natural microenvironment and functional assessment. Current microfluidic liver systems now feature improved capabilities such as gradient establishment for proper drug dose escalation, complex co‐cultures, and built‐in sensors. These systems complement and improve 3D culture models, making sure of the physiologically relevant hepatocytes for precision drug screening tests. The application of microfluidics facilitates the development of new and interesting computer‐based programs in personalized medicine, disease modeling, and drug discovery. In this regard, they are promising devices for new approaches in drug discovery and toxicology [64, 65, 66].

Emulate Inc. has presented Liver‐on‐a‐Chip. This platform recreates key aspects of the liver's complex biology and can be used to predict human response to drugs, chemicals, and cosmetics, providing better insight than conventional cell culture or animal‐based testing [54]. HepaChip has been designed by Nortis Inc, which is a microfluidic device designed for culturing hepatocytes in a physiologically relevant environment. This enables researchers to study drug metabolism and mechanisms of liver disease [67, 68]. HepatoPac by Hepregen Corp. (now part of Ascendance Biotechnology) is a micropatterned co‐culture platform that maintains functional hepatocytes along with supportive stromal cells, improving long‐term cell viability and liver‐specific functions for in vitro toxicity and drug metabolism [69]. LiverChip, the product of CN Bio, is a microphysiological system that provides long‐term in vitro culture of primary human and animal hepatocytes in a 3D format that can be used to evaluate drug safety, efficacy, and metabolism [70]. CN Bio's PhysioMimix is a microfluidics‐based organ‐on‐a‐chip (OOC) system that can replicate human and animal organ systems including the liver for advanced disease modeling and drug development applications [71, 72]. Mimetas company has provided a product named OrganoPlate. This product uses a microfluidics 3D culture platform for high‐throughput organ‐on‐a‐chip applications to culture hepatocytes along with other cell types in a microfluidic environment [73].

PHs, liver cell lines, and stem cell‐derived HLCs can be used in microfluidic platforms. Also, these platforms can be combined with several types of cells as co‐culture to establish models that closely mimic human liver physiology. In this way, valuable tools are provided for researchers in drug development. Finally, they offer a controlled condition to observe the behavior of hepatocytes in conditions that are very similar to the in vivo human liver.

## 5 Scrutiny of the Performance of Different Culture Configurations in Drug Screening

2D hepatocyte cultures have long been used for drug screening. Due to cost‐effectiveness and simplicity, 2D culture systems are well‐suited for the primary screening of numerous compounds and provide essential tests for evaluating drug metabolism and effectiveness. However, these 2D cultures poorly replicate the liver's detailed 3D architecture and cellular and cell–ECM interactions. Realistic cell–cell and cell–ECM interactions are extremely important for imitating tissue behavior in vivo, which is an essential limitation in 2D culture [69]. 2D culture systems produce valuable primitive data, but they often cannot fully model tissue function. Even, some commonly used liver cell lines exhibit different metabolic activities depending on the passage number or culture method. For example, the activity of drug‐metabolizing enzymes in cell lines such as Huh‐7 and HCC‐T varies across different passages and different lines [74]. Furthermore, a study showed that the metabolic activity of HepaRG cells also varied depending on the culture protocol. Comparing the Biopredic and Sison protocols, the Biopredic protocol maintained CYP enzyme activity (including CYP3A4, CYP1A2, and CYP2B6), drug transport function (e.g., MDR1, MRP1), and liver‐specific properties at higher levels. This demonstrates how culture conditions can affect the function of liver‐derived cell lines [75]. This makes 2D systems less reliable for in‐depth pharmacokinetic, metabolic studies and for understanding complex drug interactions. The 2D culture system's predictive power is limited; it may not always accurately predict drug responses or toxicity as they would happen in in vivo conditions. This may lead to significant controversies between the experimental, preclinical, and clinical outcomes. To improve the quality of drug screening tests, which require a more physiologically relevant approach, researchers are increasingly experimenting with other modalities choices such as 3D systems and microfluidic models [76].

In recent years, the 3D culture systems have advanced the field of drug screening. 3D culture systems emulate the in vivo microenvironment and support enhanced cell–cell and cell–ECM interactions and tissue‐like structures. This approach contributes to their superior performance in predicting drug metabolism, toxicity, and effectiveness. 3D cultures are valuable, especially in precision research such as individual drug response studies and evaluation of long‐term effects of drugs. This approach enhances the predictive accuracy of in vitro drug toxicology assays and is suggested as the preferred method in many drug screening studies [77].

However, setting up 3D cultures needs specific expertise and access to specific equipment. Researchers face more complexity within 3D culture systems about data consistency and experiment replicability. Moreover, the costs of developing 3D culture systems are high, which is due to the need for implementing specialized scaffolds, growth factors, and advanced equipment. However, their ability to mimic real organ responses in the body usually makes them a more powerful platform for these issues [78].

Microfluidic devices are leading tools in drug screening technology because they work very accurately. They are designed to control different conditions of cultures, including perfusion, oxygen levels, and shear stress, to mimic the liver's internal microenvironment accurately. Microfluidic systems are especially ideal for real‐time monitoring. They give information about how drugs metabolize, distribute in the body, and provide understanding about complex biological reactions. Microfluidics are valuable tools for researchers, and because of their accurate design and ability to mimic drug interactions in an microenvironment that is biologically similar to the human body, they could be used in controlled conditions. Hence, microfluidic platforms make it possible to investigate drug kinetics, drug interactions, and complicated cellular responses in vitro [79].

Microfluidic devices are sophisticated tools for hepatocyte‐based drug screening. However, microfluidic systems have several challenges that cause many limitations in their application. Setting up microfluidic systems is technically complex and usually needs someone with bioengineering skills. It is also an expensive technology on the beginning setups, especially for custom‐made ones. Additionally, microfluidic systems need regular maintenance, like routine cleaning for keeping the microchannels functional [80, 81].

**FIGURE 3:**
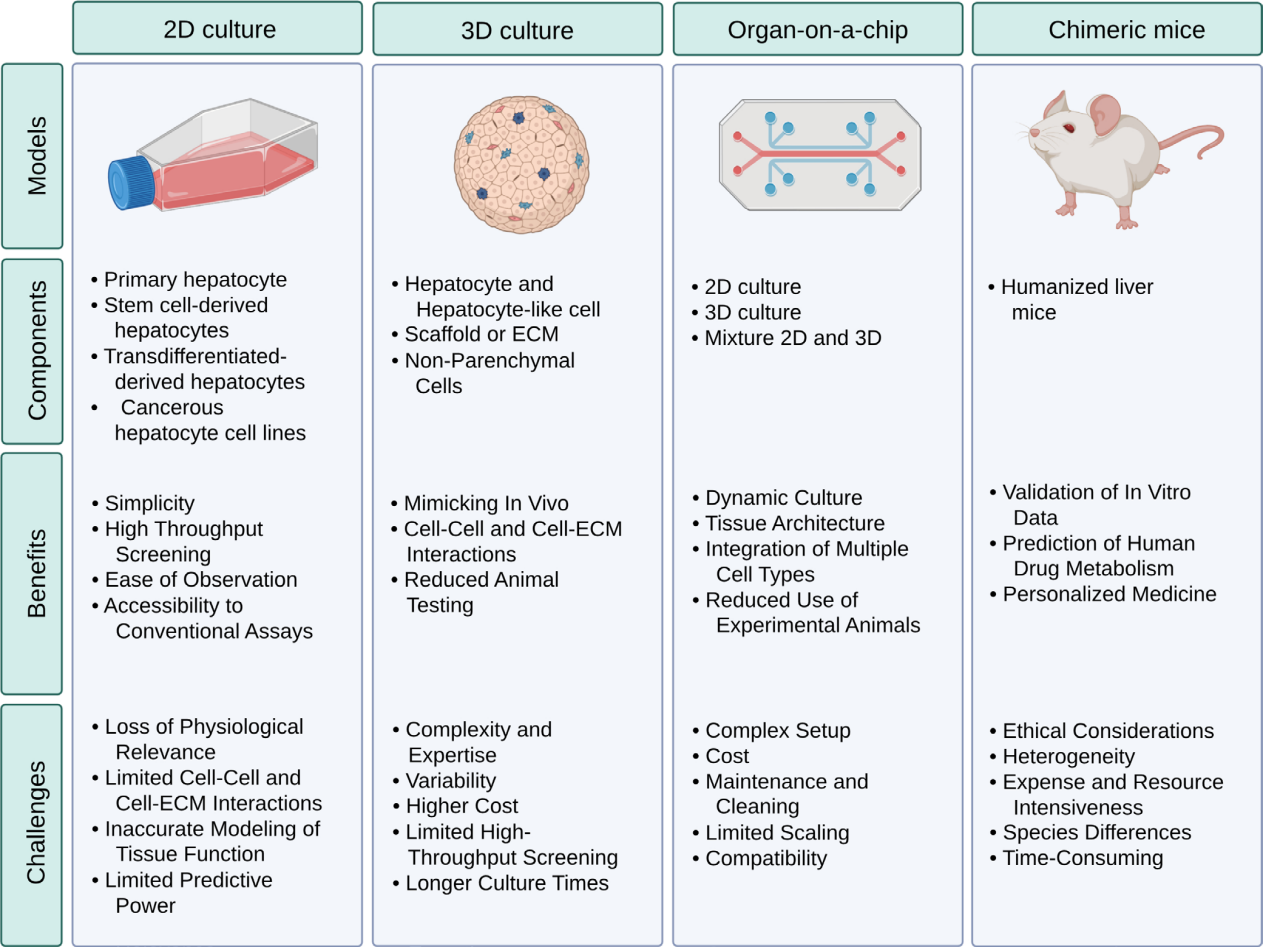
Investigation of different culture configurations based on hepatocyte cells in drug discovery and toxicology.

It is difficult to scale up microfluidic‐based experiments. Microfluidic systems might not be compatible with all cell types or tissues, which could limit their use. Also, other issues still exist like cost, complexity, and the feasibility of their broad use (Figure 3) [81].

## 6 Innovative Functional Cell Sources and Culture Configurations

**FIGURE 4:**
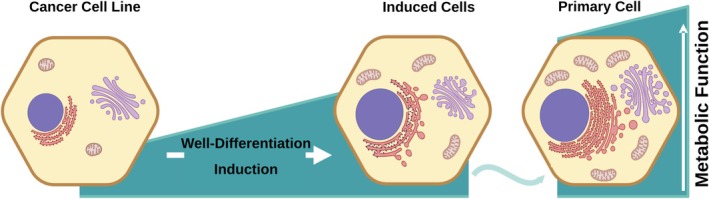
Use of hepatoma cells with enhanced physiological function as an innovative tool for drug discovery and toxicological studies. Well‐differentiation induction improves the physiological function of hepatoma cells.

Using 3D cultures and microfluidics systems is a very promising configuration to mimic in vivo conditions. 3D cultures and organoids generate a physiologically relevant microenvironment for culture cells. Due to cell–cell and cell–ECM interactions, cells in these culture configurations interact more naturally, which causes mimicking the complexity of in vivo tissue structures. Using these improved culture methods with HLCs originating from modified hepatoma cells offers an ideal tool for a more realistic and reliable drug screening platform. This could speed up drug discovery, reducing research and development costs, and reducing the ethical issues associated with animal models (Figure 4) [82, 83, 84].

## 7 Conclusion

In conclusion, the need for available, reliable, and rapid ex vivo drug screening methods has led us to explore innovative platforms to mimic the in vivo microenvironment. Selecting the most appropriate cell type and optimized co‐culture configuration is critical. Improving the metabolic performance of hepatoma cells could be an innovative strategy in developing novel platforms in ADME‐Tox assays [78, 79].

Furthermore, the use of 3D culture and microfluidic systems provides a realistic achievement for in vivo imitation of drug screening procedures. These advanced cell culture techniques simulate the liver microenvironment and cell–cell interactions. Therefore, they can enable us to perform more accurate drug metabolism and hepatotoxicity assessments. Integration of manipulated hepatoma cell lines and advanced 3D co‐culture methodologies provides more reliable tools in drug screening and saves money and time as well as minimizes ethical concerns related to animal testing. These synergy and combined approaches are leading advances in drug discovery, making precise and rapid lab assessments for new drugs, which will lead to safe and effective medications in the future [78, 79].

## Author Contributions

Z.H. and M.V. contributed to the conception, design, and drafting of the manuscript. B.S. and M.N. were involved in writing, editing, and proofreading of the manuscript. All authors approved the final version for submission.

## Conflicts of Interest

The authors declare no conflicts of interest.

## Data Availability

The authors have nothing to report.
